# Minimally Invasive Direct Lateral Transpsoas Approach for the Resection of a Lumbar Plexus Schwannoma: Technique Report

**DOI:** 10.1055/s-0036-1587692

**Published:** 2016-08-06

**Authors:** Carolina G. Benjamin, Eric K. Oermann, J. Alexander Thomas, Casey T. Distaso, Faheem A. Sandhu

**Affiliations:** 1 Georgetown Medical School, Washington, District of Columbia; 2 Department of Neurosurgery, Medstar Georgetown University Hospital, Washington, District of Columbia

**Keywords:** lateral, retroperitoneal, transpsoas, minimally invasive, lumbar plexus tumors, schwannoma

## Abstract

**Objective** Traditional techniques for resection of lumbar plexus tumors have been associated with approach-related morbidity. We describe a case utilizing a minimally invasive transpsoas lateral access approach to resect a retroperitoneal tumor of the lumbar plexus.

**Methods** We report a case with an extradural retroperitoneal schwannoma of the L4 nerve root that was treated with a minimally invasive direct lateral transpsoas approach using atraumatic tissue dilators and an expandable tubular retractor. The use of directional and continuous electromyographic monitoring was critical in locating the plexus and positioning the retractor immediately anterior to the tumor.

**Results** The patient tolerated the procedure well without postoperative complications. The operative approach was direct and intraoperative blood loss was negligible. The patient demonstrated improved left leg strength and ambulation and resolution of paresthesias.

**Conclusions** A minimally invasive direct lateral transpsoas access approach is an effective technique to safely and adequately resect extradural retroperitoneal lumbar plexus tumors.

Lumbar plexus schwannomas are indolent, benign tumors usually arising from dorsal nerve roots that manifest clinically with radicular pain, weakness, and paresthesia depending on the level of the tumor. 1 2 3 4 Although case reports have shown that some benign schwannomas are hypointense on T1-weighted images and hyperintense on T2-weighted images, there are no unifying patterns on magnetic resonance imaging (MRI) that characterize these tumors. 5 Traditional surgical therapies for such tumors include a laparoscopic or mini–open anterior transperitoneal approach, a posterior midline approach, or a paraspinal approach. Based on tumor size, location, and characteristics, some of these approaches may require concomitant hemilaminectomy and facetectomy with possible arthrodesis. 2 6 7 8 9 10 11 12

Minimally invasive techniques offer an advantage over traditional surgical methods by reducing soft tissue trauma and blood loss, thus lessening approach-associated morbidity. 7 8 13 A lateral transpsoas approach, as popularized in lateral interbody fusion surgery, is the most direct technique to approach retroperitoneal lumbar plexus tumors and should help to reduce postoperative pain and hospital stays and expedite the return to activities of daily living. 6 7 8 13 14 15 Furthermore, such an approach precludes the need for an access surgeon. 13

In this report, we describe a direct lateral minimally invasive approach to the retroperitoneum utilizing a tubular retractor and directional electromyography (EMG) for complete resection of an L4 nerve root schwannoma distal to the dorsal root ganglia in the psoas muscle.

## Case Report

**Fig. 1:**
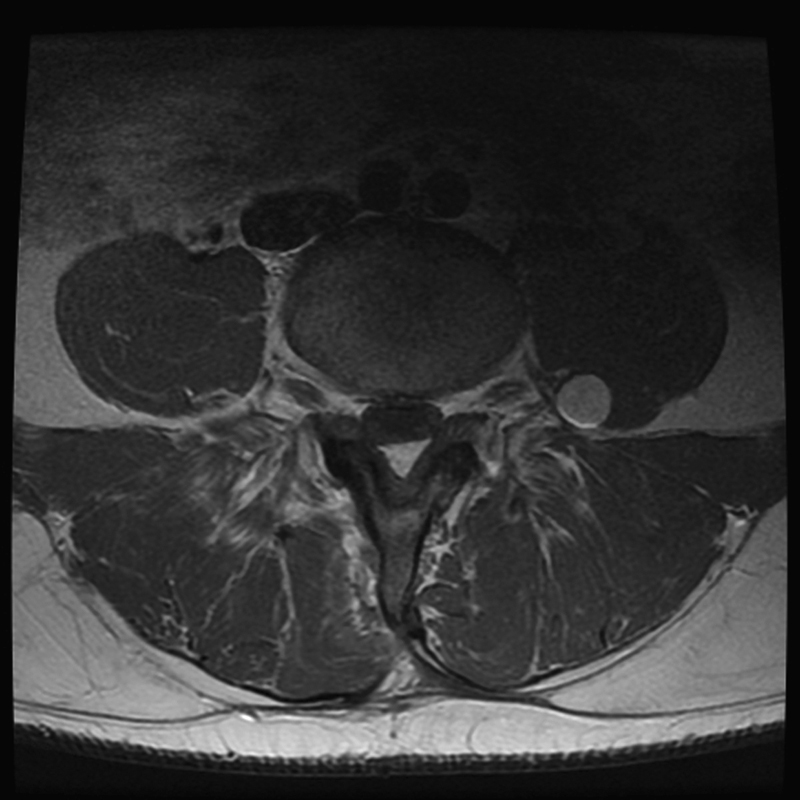
Preoperative axial magnetic resonance imaging with contrast demonstrating enhancing mass in the left psoas muscle.

A 38-year-old man presented with a 2-year history of lumbar pain radiating to the toes bilaterally and progressive motor weakness in his feet. In 2008, the patient underwent an L4–L5 laminectomy and diskectomy at an outside institution for his low back and leg pain without clinical improvement. The patient had progression of left leg pain associated with weakness over the next 10 months. A lumbar MRI done during workup at an outside institution revealed central disk herniation and stenosis at the L4–L5 level and a 1-cm enhancing nodule of the left L4 nerve root distal to the dorsal root ganglion. The treating surgeon ignored the presence of the tumor and performed an L4 laminectomy and L4–L5 posterolateral arthrodesis. The patient experienced some improvement of back pain after surgery but not surprisingly continued to have severe left leg pain and weakness. He presented to our institution with increased severity of pain, weakness, and paresthesias in the left leg. Neurologic examination revealed motor deficit (4/5 strength) in his left quadriceps and dorsiflexion. He had decreased sensation to light touch and pinprick in a left L4 distribution and diminished left patellar reflex, and he required a cane to assist with ambulation. Repeat MRI studies ( Fig. 1 ) demonstrated an interval increase in the size of the tumor of the L4 nerve root when compared with a study done 1 year prior. The location of the tumor clearly correlated with his persistent symptoms, and a surgical resection was planned.

**Fig. 2:**
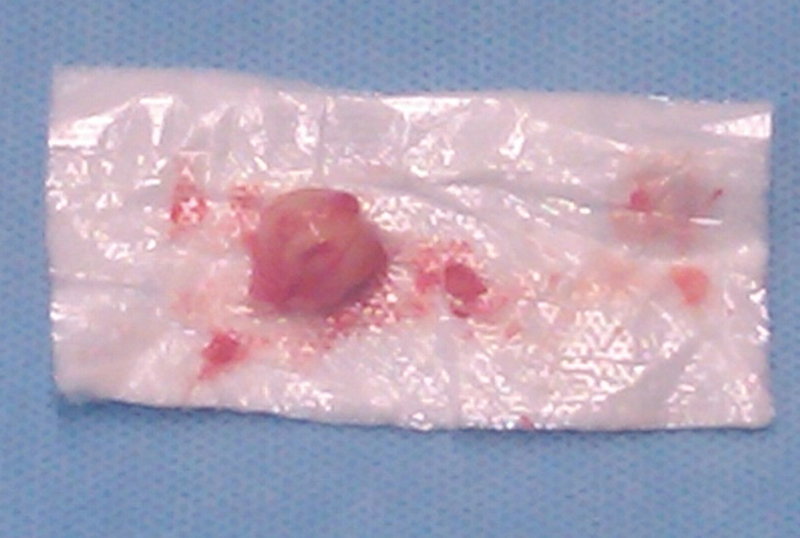
Photograph of gross total tumor resected from nerve root consistent with schwannoma.

**Fig. 3:**
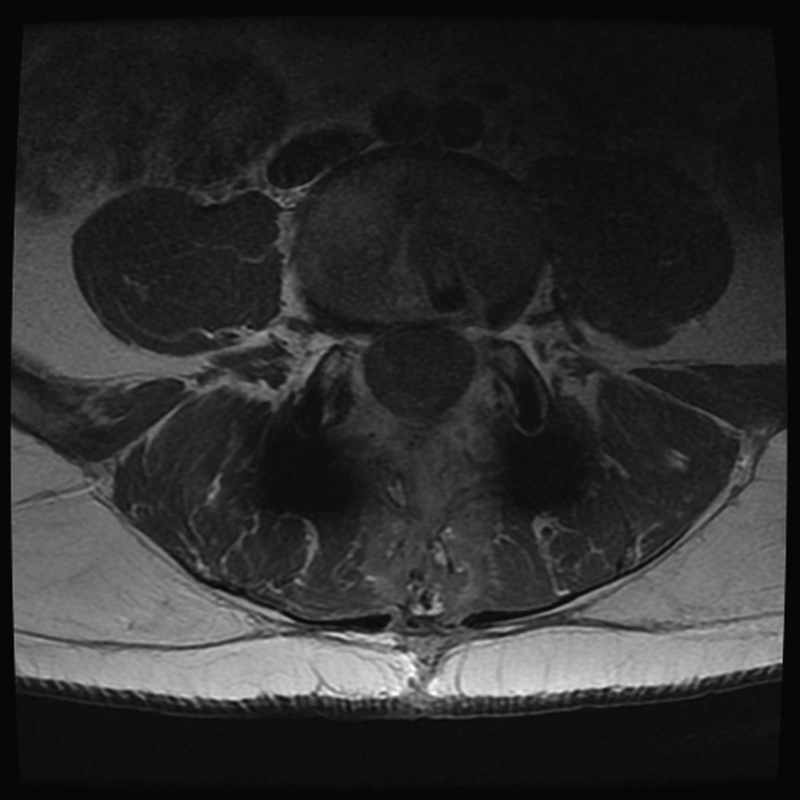
Axial magnetic resonance imaging with contrast 1 year after tumor resection demonstrating no evidence of tumor recurrence. The patient had undergone L4–L5 fusion elsewhere prior to tumor resection.

A minimally invasive resection of the tumor via a retroperitoneal transpsoas approach was performed. Following access to the retroperitoneal space, an initial dilator (MaXcess System, NuVasive, Inc., San Diego, California) was used to traverse the psoas muscle using continuous and directional EMG monitoring, and then was docked onto the posterior aspect of the L4–L5 disk. Care was taken to get as close to the nerve root as possible with the dilators positioned just anterior to the nerve as mapped using the directional stimulation located in the tip of the dilators. The working retractor was then placed so that the expandable blades faced posteriorly to allow direct visualization of the tumor. A nerve probe was used to locate the position of the L4 nerve root, which was posterior to the retractor. After a small amount of muscle dissection, the tumor and lumbar plexus were quickly encountered. Using a microsurgical technique, the tumor was circumferentially isolated, and intercapsular dissection was performed until the tumor was fully delineated. Complete tumor removal and preservation of the parent nerve root were achieved ( Fig. 2 ). Neurophysiologic monitoring was unchanged over the course of the surgical procedure. Operative time was 65 minutes with minimal blood loss. Pathologic examination of the specimen confirmed the diagnosis of a benign schwannoma. Neurologic examination postoperatively demonstrated immediate improvement in quadriceps and dorsiflexion strength (4 + /5) and resolution of paresthesias. The patient did experience new dysesthesia in an L4 distribution that were quite severe initially but improved with time and the administration of pregabalin and steroids. The patient had an uncomplicated hospital course and was discharged on postoperative day 5. At 1-year follow-up, his strength and ambulation had improved; the left leg dysesthesia was stable and tolerable with the use of pregabalin. A postoperative MRI scan demonstrated complete resection of the schwannoma without any evidence of recurrence ( Fig. 3 ).

## Discussion

Spinal schwannomas are benign peripheral nerve sheath tumors that arise primarily from the lumbosacral region and produce the characteristic symptoms of lower extremity sensory and motor deficits. 2 3 7 15 These tumors can be either intradural, extradural, or both. Rarely, these tumors arise within the lumbar plexus in the retroperitoneum as in this case. The location of the tumor as well as the nerve root from which the tumor arises determines the clinical symptoms experienced by the patient. 2 3 It also becomes an important factor in planning surgical intervention that involves complete surgical resection with preservation of the integrity of the parent nerve when possible. 1 16 The shape, size, and location of these tumors as seen on CT and MRI are critical factors when choosing the surgical approach best suited for effective isolation and resection of the tumor. CT characteristics of benign retroperitoneal schwannomas include smooth tumor margins, evidence of necrosis and hemorrhage, and, often, cystic changes. 17 Although MRI has become an important tool in making a presumptive diagnosis and in surgical planning, there are no unique characteristics of benign retroperitoneal schwannomas on MRI that can guide treatment strategies accordingly. 5

The most traditional method for resection of lumbar plexus tumors is an open anterior retroperitoneal approach with or without laparoscopy. This approach attempts to achieve a complete resection while preventing large-scale hemorrhage by isolating the vasculature before proceeding with the enucleation and removal of the tumor. 16 In cases where complete resection cannot be achieved, hemorrhages up to 2.5 L have been described in the literature. 10 Laparoscopy attempts this same anterior technique in a less invasive manner to improve postoperative quality of life. 17 Although it can be done successfully, it is technically demanding, and patients have to be chosen carefully to prevent complications and damage to nearby vital structures. Other approaches include a paraspinal approach or midline posterior approach requiring total facetectomy with the need for instrumented arthrodesis. These approaches, too, have several well-known risks including hemorrhage, nerve injury, and muscle injury. 6 8 9 10 11 13 18 19 20 Additionally, such approaches require a prolonged hospitalization period for appropriate recovery. This case report describes a technique adapted to minimize these risks. Phan and Mobbs described an anterior retroperitoneal approach that allows direct access without the need for laminectomy or facetectomy, aimed at decreasing the risks of traditional posterior approaches. 21 Although this approach may be considered minimally invasive, we feel the approach may be simplified further with the use of a tubular/expandable retractor and the use of direction EMG, which provides additional safety during the initial dissection of the psoas muscle. Theoretically, this approach should in turn minimize dissection through the psoas muscle. Also, use of a tubular retractor affords smaller incisions, reducing potential complications including incisional hernia.

The use of a minimally invasive lateral transpsoas approach for the resection of a lumbar plexus tumor is a recent application of a technique commonly used for thoracolumbar fusion procedures. 6 7 13 14 15 A major advantage of this technique is the use of directional and continuous EMG monitoring during the approach that allows for the most direct and least traumatic exposure of the tumor in comparison to other conventional approaches, even when laparoscopy is used. 13 17 Decreased operative time and minimal tissue trauma lead to faster recovery rates and shorter hospitalizations. 7

Lu et al and Shah et al described minimally invasive posterolateral techniques to remove an extradural intraradicular lumbar schwannoma using tubular retractors. 9 19 22 Both groups reported successful treatment outcomes, substantiating the role of minimally invasive procedures for the removal of extradural foraminal tumors. However, there are certain key differences between our technique and the ones described by other authors. First, the use of a transpsoas approach has some advantages over a posterolateral approach. It provides a more direct path to the tumor and avoids removal of the facet and subsequent arthrodesis, which would be required for intraforaminal tumors. Even in the absence of facetectomy, posterolateral approaches are associated with greater morbidity due to increased soft tissue trauma to posterior stabilizing muscles. 13 Another advantage of our technique is the use of an expandable tubular retractor that allows for better visualization of the surgical field and a larger working area than a closed tubular retractor, as used by Shah et al and Lee and Srikantha. 19 23 A closed tubular retractor can limit exposure that is critical when attempting to completely remove a tumor using microsurgical dissection. Gonçalves et al utilized an expandable tubular retractor system and reported successful removal of an extradural far lateral lumbar schwannoma. 24 This result, as well as our own, corroborates the efficacy of minimally invasive approaches that utilize an expandable retractor.

Finally, our technique employs continuous EMG monitoring to provide an additional safety measure throughout the procedure to prevent iatrogenic nerve damage. Similar directional EMG monitoring, located at one point on the tip of the dilators, was additionally employed to localize the nerve sheath tumor itself following successful navigation of the psoas muscle.

The lateral transpsoas access approach, like any other procedure, is not without risk. A possible complication of the lateral transpsoas approach is motor and sensory nerve injury when traversing the lumbosacral plexus with the dilator or during retractor positioning over the disk space. 14 15 To avoid such complications, directional and continuous EMG monitoring should be used to delineate the lumbosacral plexus, and fluoroscopy should be used to locate the disk space as was done in this case.

## Conclusion

We describe a new use of the direct lateral transpsoas approach to the lumbar spine in the treatment of a retroperitoneal lumbar plexus schwannoma. This approach can be superior to conventional approaches if the tumor is extraforaminal and distal to the dorsal root ganglia. As demonstrated in this case, continuous intraoperative EMG monitoring can be used as both a means of avoiding nerve injury while transversing the psoas when directional EMG stimulation was employed and as a means of localizing the schwannoma itself prior to resection. Careful evaluation of each case is necessary to determine if a direct lateral access approach is appropriate for successful surgical resection.
